# Glucocorticoid receptor isoforms direct distinct mitochondrial programs to regulate ATP production

**DOI:** 10.1038/srep26419

**Published:** 2016-05-26

**Authors:** David J. Morgan, Toryn M. Poolman, Andrew J. K. Williamson, Zichen Wang, Neil R. Clark, Avi Ma’ayan, Anthony D. Whetton, Andrew Brass, Laura C. Matthews, David W. Ray

**Affiliations:** 1 School of Computer Sciences, University of Manchester, Kilburn Building, Oxford Road, Manchester, Uk, M13 9PL; 2 Faculty of Medical and Human Sciences, University of Manchester, AV Hill Building, Oxford Road, Manchester, UK, M13 9PT; 3 Manchester Centre for Nuclear Hormone Research in Disease, University of Manchester, AV Hill Building, Oxford Road, Manchester, UK, M13 9PT; 4 Manchester Academic Health Sciences Centre, University of Manchester, AV Hill Building, Oxford Road, Manchester, UK, M13 9PT; 5 Department of Pharmacology and Systems Therapeutics, Icahn School of Medicine at Mount Sinai, One Gustave L. Levy Place, Box 1603, New York, NY 10029, USA; 6 Stoller Biomarker Discovery Centre, University of Manchester, Wolfson Molecular Imaging Centre, Palatine Road, Manchester, UK, M20 3LJ; 7 Faculty of Life Sciences, University of Manchester, AV Hill Building, Oxford Road, Manchester, UK, M13 9PT.; 8 Faculty of Medicine and Health, University of Leeds, Wellcome Trust Brenner Building, St James’s University Hospital, Leeds, UK, LS9 7TF.

## Abstract

The glucocorticoid receptor (GR), a nuclear receptor and major drug target, has a highly conserved minor splice variant, GRγ, which differs by a single arginine within the DNA binding domain. GRγ, which comprises 10% of all GR transcripts, is constitutively expressed and tightly conserved through mammalian evolution, suggesting an important non-redundant role. However, to date no specific role for GRγ has been reported. We discovered significant differences in subcellular localisation, and nuclear-cytoplasmic shuttling in response to ligand. In addition the GRγ transcriptome and protein interactome was distinct, and with a gene ontology signal for mitochondrial regulation which was confirmed using Seahorse technology. We propose that evolutionary conservation of the single additional arginine in GRγ is driven by a distinct, non-redundant functional profile, including regulation of mitochondrial function.

Glucocorticoids (Gc) exert diverse effects on cell fate, energy metabolism, and immune regulation through the glucocorticoid receptor (GR), a member of the nuclear receptor superfamily. In its unliganded state GR is predominantly cytoplasmic, sequestered in a multiprotein complex that includes immunophilins and heat shock proteins. Ligand binding induces a conformational change in the receptor, which is accompanied by rapid post-translational modification of the GR, most notably by phosphorylation. The transformed GR is then released from the multiprotein complex, rapidly translocates to the nucleus and binds to cis-elements to regulate gene expression.

A feature of all nuclear receptors, including GR, is a modular structure comprising an N-terminal modulating domain, a C-terminal ligand binding domain and a central DNA binding domain (DBD). The DBD is critically important for directing sequence specific DNA binding, it lies adjacent to a nuclear localisation signal, and also is an important protein interaction surface, coordinating the recruitment of proteins to GR complexes. Therefore modification of the DBD may alter target gene selection, nucleocytoplasmic shuttling and protein-protein interactions.

GRα is the most abundant isoform, accounting for 90% of GR transcripts across all tissues and is considered the primary mediator of Gc action *in vivo*. The GRγ isoform is conserved through mammalian evolution, constituting approximately 10% of GR transcript abundance in all tissues^1^,^2^, but its specific function remains elusive. GRγ was originally thought to arise from species variation^3^, or mutation^4^,^5^, but is now recognised to be a constitutive splice variant^2^. In some reports altered GRγ expression has been observed in association with altered Gc responses^6^,^7^.

Alternative splicing between exons 3 and 4, which together encode the DBD, produces the GRγ isoform. In fish, alternate exon 3 and 4 splicing incorporates a distinct exon, adding 9 unique amino acids between the two zinc fingers, but in mammals, the GRγ isoform has a single additional arginine. Structure-function studies of GRγ identified disruption of the lever arm between the two alpha helices (Fig. 1A), and as a consequence an alteration in the DNA sequence binding preference. This results in a difference in the transcriptional regulation of a subset of genes, such as BIRC3 and SDPR, whilst the activation of some genes by GRα and GRγ, such as FKBP5, remains similar^8^. These differences are not due to altered DNA binding affinity or GR occupancy at the target genes which suggests that the altered conformation of the lever arm of GRγ interprets an allosteric signal from the DNA differently, resulting in a functional effect^8^. Indeed, more recent ChIP-seq studies have demonstrated that GRγ has different sequence specificity when compared with GRα^9^.

For GRγ to be conserved through mammalian evolution it is likely that some positive selective pressure would be required, implying a specific, non-redundant function. Here we define a distinct GRγ driven signalling network including identification of GRγ specific subcellular trafficking, target gene selection, and engagement of interacting proteins. Both transcriptome, and protein interactome data suggested a role in for GRγ in directing mitochondrial function, and indeed GRγ expression increased mitochondrial mass, basal respiration, and ATP generation.

## Results

**Figure 1:**
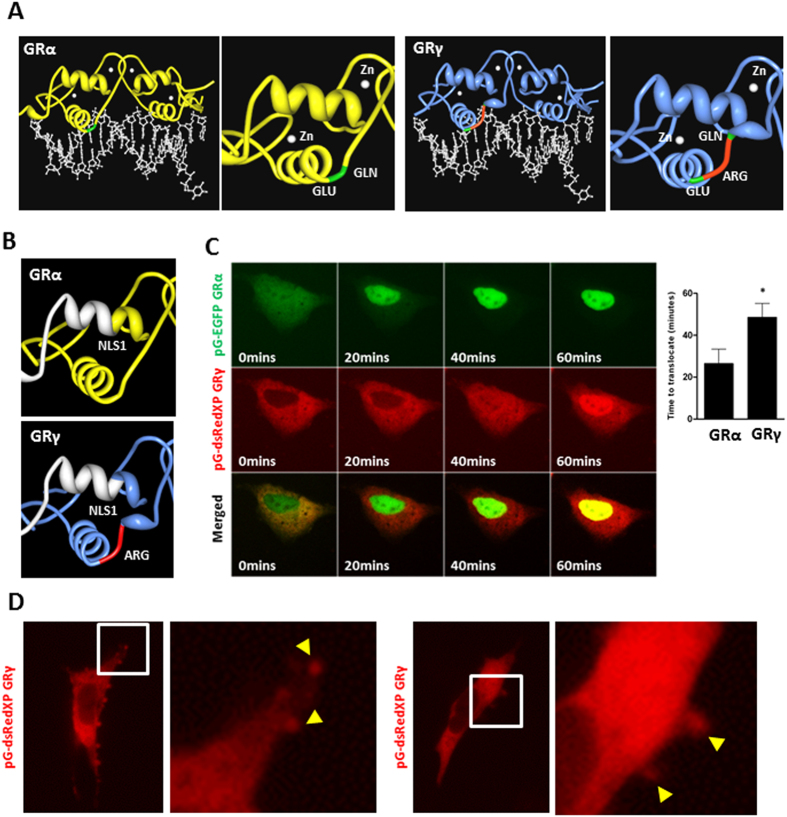
Kinetics of GRα and GRγ activation. (**A**) Protein Workshop was used to illustrate conformational differences of GR isoforms bound to FKBP5 promoter (PDB ID 3G6U and 3G6T^8^). GRγ (blue) differs from GRα (yellow) by a single arginine residue inserted in the DBD, which is a DNA and protein interaction surface. The additional arginine in the DBD is highlighted in red, with adjacent amino acids shown in green. (**B**) Protein Workshop was used to illustrate the proximity of the additional arginine to NLS1, shown in white. (**C**) Cells were co-transfected with pG-EGFP GRα and pG-dsRedXP GRγ, cultured in charcoal stripped serum, treated with 100 nM dexamethasone and imaged using time-lapse microscopy. An example of GRα (green) and GRγ (red) expression in the same cell is shown. Nuclear translocation from multiples cells were quantified. Graphs (mean +/− SD) are representative of three independent, and a total of 71 cells were analysed. Samples were compared with an unpaired, two-tailed Student’s t test (*P < 0.05). (**D**) Cells were transfected with pG-dsRedXP GRγ, cultured in charcoal stripped serum and imaged using time-lapse microscopy. Expanded regions are highlighted with white boxes. Arrows indicate GRγ at membrane ruffles.

The additional arginine of GRγ lies close to the major nuclear localisation signal (NLS1), and may perturb nucleocytoplasmic shuttling (Fig. 1B). To test this, fluorophore tagged GR isoforms were co-expressed. There was a clear difference in isoform distribution, with GRγ being more cytoplasmic under ligand-free condition, and showing significantly delayed rates of ligand-induced nuclear import (Fig. 1C, movie S1).

Further live cell analysis revealed striking, organised assembly of the GRγ isoform at sites of membrane ruffling, best seen in the accompanying video (Fig. 1D, movie S2). A recent model suggested that nuclear receptors serve as molecular “ferryboats”^10^, which were required to traffic to the plasma membrane in order to become activated. The membrane proximal location of the GRγ isoform suggests that this isoform may preferentially respond to lower ligand concentrations and therefore offers an ideal system to test the “ferryboat” theory. However, we found no difference in Gc sensitivity between the GR isoforms (Fig. S1A,B, movie S3).

**Figure 2:**
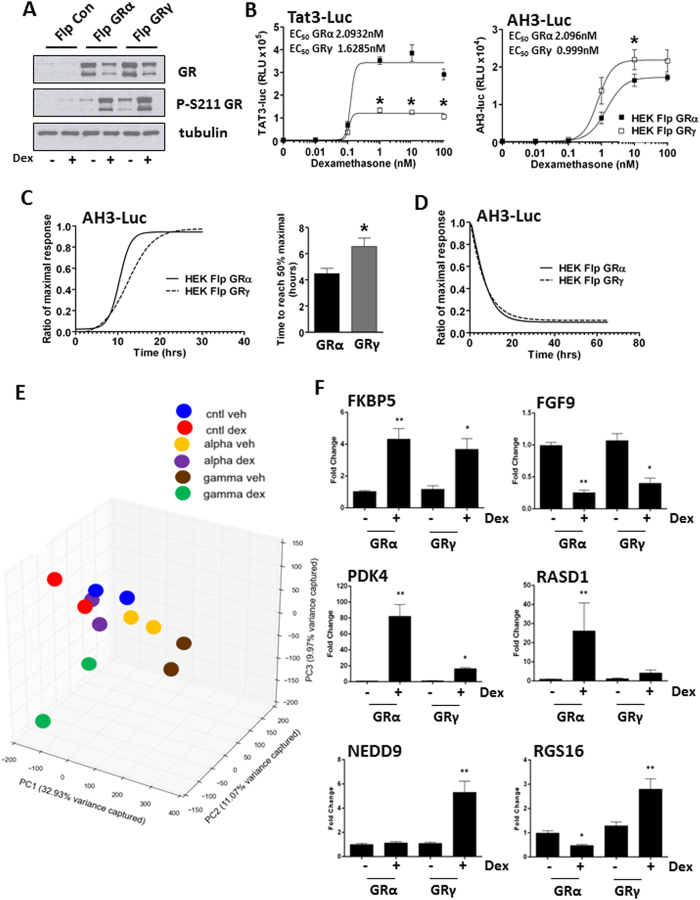
GRα and GRγ transcriptome profiling. (**A**) HEK Flp, HEK-FlpGRα and HEK-FlpGRγ cells were treated with 100 nM dexamethasone for 1 hour then immunoblotted for total GR and phosphorylated serine 211 GR. Tubulin is shown as a loading control. (**B**) HEK-FlpGRα and HEK-FlpGRγ cells were transfected with 2 μg of either TAT3-luc or AH3-luc. Cells were treated for 16 hours with varying concentrations of dexamethasone, lysed and then analysed by luciferase assay. EC50 values are indicated. Samples compared by 2 way ANOVA, (*P < 0.05). (**C**) HEK-FlpGRα and HEK-FlpGRγ cells were transfected with AH3-Luc, treated with 100 nM dexamethasone and the production of luciferase monitored for twenty four hours. Production of luciferase is displayed as a ratio of the maximal response. Graphs (mean ± SD) combines data three independent experiments. Samples were compared with an unpaired, two-tailed Student’s t test (*P < 0.05). (**D**) Cells from (**C**) were washed, to remove Gc and the production of luciferase measured for a further 66 hours. HEK Flp, HEK-FlpGRα and HEK-FlpGRγ cells were treated with 100 nM dexamethasone for 4 hours, RNA extracted and duplicate samples subjected to microarray analysis. (**E**) Principal Component Analysis (PCA) of the gene expression data. Samples profiled by microarray are plotted in the first three principal components space, coloured by condition. Percentages of total variance by each principal component are indicated in the axis labels. (**F**) HEK-FlpGRα and HEK-FlpGRγ cells were treated with 100 nM dexamethasone for 4 hrs, RNA extracted and analysed by qPCR. Targets regulated by both GRα and GRγ (FKBP5, FGF9), or specifically by GRα (PDK4, RASD1) or GRγ (NEDD9, RGS16) are shown. Graphs (mean ± SD) combine data from three separate experiments and display fold change over vehicle treated control. Stats were performed by one-way ANOVA. Values considered significant when p-value is <0.05. *significant compared to vehicle treated controls, **significant when compared to all samples.

We next compared overexpression of GRα and GRγ in HEK293 cells, which are deficient in endogenous functional GR, using Flp recombinase technology (Fig. S1C). This permitted stable expression of either GRα or GRγ with matched integration site and the same level of expression (Fig. 2A). We used the GR isoform specific reporter constructs CGT-luc and KLK3-luc, to confirm the functional acquisition of stable GRα and GRγ expressing cells (Fig. S1D). We also found differences in maximal transactivation of two further reporter genes, emphasising the importance of DNA target sequence (Fig. 2B).

As the AH3-luc reporter had similar EC50 and maximal response to both GR isoforms, we used this system to measure transactivation kinetics. GRγ transactivation showed a slower onset of transactivation than GRα (Fig. 2C), compatible with the slower rate of translocation, while the decay in transactivation following ligand withdrawal occurred at similar rates for the two isoforms (Fig. 2D).

We next mapped isoform specific Gc targets using gene expression cDNA microarrays. To eliminate effects arising from kinetic differences between the two isoforms, we profiled transcriptomes of GRα and GRγ stable FlpIn cells after four hours Dexamethasone treatment.

Principle component analysis (PCA) shows that duplicate samples cluster together, that both GRα and GRγ shift global gene expression from the control, and that Dexamethasone treatment induces a shift in global gene expression, with greater change induced by GRγ (Fig. 2E). Using the Characteristic Direction method^11^ we next identified differentially expressed genes between the control, GRα and GRγ cells (Supp. Data File 1) and identified a panel of differentially expressed genes (Figs 2F and S3). Furthermore, we successfully validated these Gc regulated genes in independently derived stable cell lines, and in tetracycline inducible cells (T-RexFlpIn) (Figs S4 and S5).

**Figure 3:**
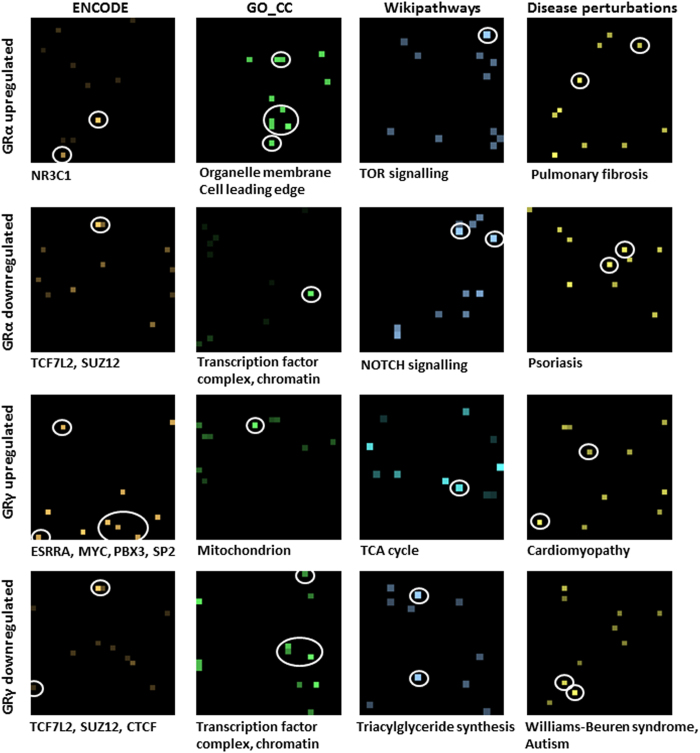
GRα and GRγ transcriptome profiling. Canvases showing the enriched biological terms for the lists of differentially expressed genes in GRα or GRγ cells after activation with Dex (vertical plane). Each canvas represents a specific gene-set library (ENCODE, GO_CC, Wikipathways, Disease perturbations from GEO), where each square on the canvas represents a single gene set (horizontal plane). The brightness of the squares on the canvas indicates the significance of the enrichment with the gene-set. Squares corresponding to the terms listed underneath each canvas are circled.

Enrichment analyses of the differentially expressed genes was performed with Enrichr^12^ using the gene set libraries from ENCODE^13^,^14^, Gene Ontology (GO) cellular component^15^, Wikipathways^16^,^17^ and disease perturbations from GEO^18^,^19^. This enrichment analysis revealed distinct roles for the two GR isoforms (Supp. Table 1). Genes repressed by GRγ had significant overlap with target genes of TCF7L2 (ENCODE, Fisher exact p-value = 3.1e-21) and CTCF (ENCODE, p-value = 2.7e-6) as well as enrichment for triacylglyceride synthesis (Wikipathways, p-value = 5.9e-4). Genes induced by GRγ were enriched for components of the mitochondrion (GO_CC, p-value = 7.5e-4) and linked with the TCA cycle (Wikipathways, p-value = 8.6e-3). Importantly, although GRα and GRγ co-regulated some target genes, our transactivated GRα-specific regulated gene set was highly correlated with a previous GR ChIP-seq study (ENCODE, p-values < 1e-8), whereas the GRγ-specific regulated gene set did not (Figs 3 and S2), supporting distinct cistromes. These isoform differences are further supported by finding that GRα genes are associated with pulmonary fibrosis and psoriasis (Disease perturbations, p-value = 3.0e-10, 2.2e-12 respectively), while GRγ genes associate with cardiomyopathy and Williams-Beuren syndrome (p-value = 2.0e-7, 2.1e-8 respectively, Fig. 3).

Unexpectedly, we also found significant differences in the transcriptional profiles between GRα and GRγ in the unliganded state, suggesting a differential ligand-independent role for the two isoforms (Fig. S6A). Although we were able to replicate this finding in independently derived stable FlpIn cell lines, we found no ligand-independent GRγ transcripts using T-Rex FlpIn cells (Fig. S6B), suggesting a chronic GRγ-dependent cell adaptation, not seen over the shorter time span of tetracycline induction in the TRex system, or confounding induced by tetracycline.

**Figure 4:**
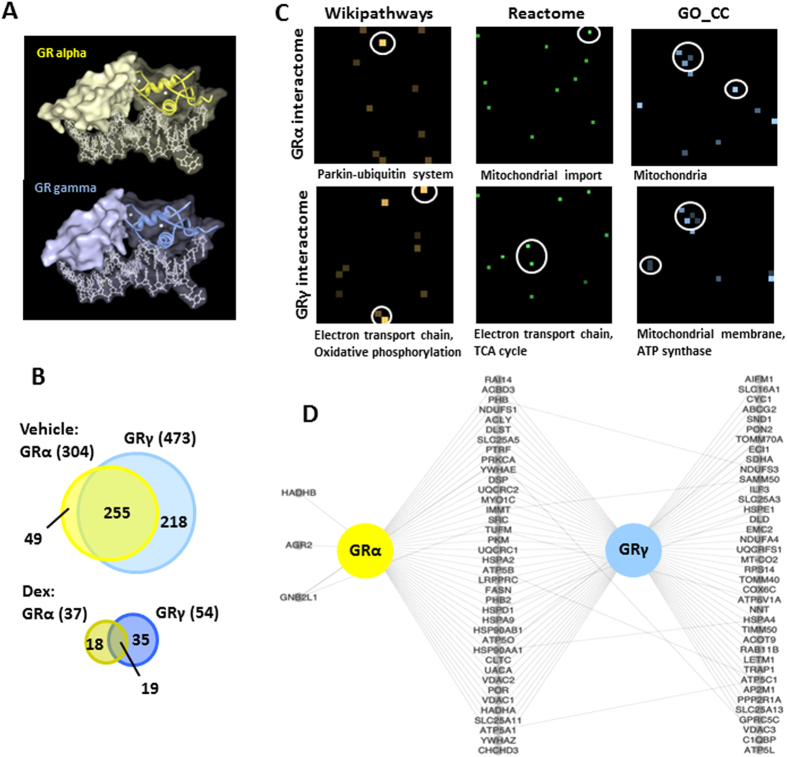
GRα and GRγ interacting proteins. (**A**) Protein Workshop was used to generate a partial surface fill of GR isoforms bound to FKBP5 promoter (PDB ID 3G6U and 3G6T^8^). (**B**) GRα and GRγ interacting proteins were determined by proteomics using a HaloTag system. GRα and GRγ expressing cells were treated with vehicle or 100 nM dexamethasone for 1 hour, GR-protein complexes purified and determined by MS/MS. Venn diagrams summarise unliganded and liganded GR interactomes. (**C**) Canvases showing the enriched biological terms for the GRα or GRγ identified protein interactions by IP-MS. Each canvas represents a gene-set library (Wikipathways, Reactome, GO_CC) where each square on the canvas represents a single gene set. The brightness of the squares on each canvas indicates the significance of the enrichment with the gene-set. Squares corresponding to the terms listed underneath each canvas are circled. (**D**) A network showing the protein interactors identified in this study for GRα or GRγ that are also labeled as mitochondria using the GO cellular component ontology.

The DBD is an important protein interaction surface (Fig. 4A). To further discriminate between the two isoforms we next considered the protein interactions they engage. We purified GRα and GRγ with their putative interacting proteins using a Halo-tag expression system. Using data derived from two independent mass spec (M/S) analyses, we identified a total of 868 GR interacting proteins (Supp. Data File 2). Of these, 67 protein interactions interacted specifically with GRα whereas 253 were specific to GRγ (Figs 4B and S7). We identified significant differences between protein partners of the two isoforms. Constitutive interactions were similar between the two isoforms, predominantly heat shock proteins, with correlation to previous low content studies (Fig. S8). The greatest differences in interaction profile were evident in vehicle treated cells with 49 interacting proteins unique to GRα and 218 interacting proteins specific to GRγ (Fig. 4B), again a likely reflection of different intracellular distributions in the unliganded state.

Enrichment analysis (Fig. 4C and Supp. Table 1) using datasets from Wikipathways^16^,^17^, Reactome^20^,^21^ and Gene Ontology (GO) cellular component^15^ suggested association of GRγ with the electron transport chain and oxidative phosphorylation (Wikipathways, p-value = 1.7e-5, 2.8e-3), TCA cycle (Reactome, p-value = 9.4e-5), and components of the mitochondrial membrane (GO_CC, p-value = 6.8e-10). More detailed analysis of the GR interacting proteins associated specifically with mitochondria revealed shared GRα/GRγ and unique GRγ mitochondrial-specific interactions (Fig. 4D). This is compatible with the previously reported mitochondrial localisation of GR (http://www.genecards.org/NR3C1).

**Figure 5:**
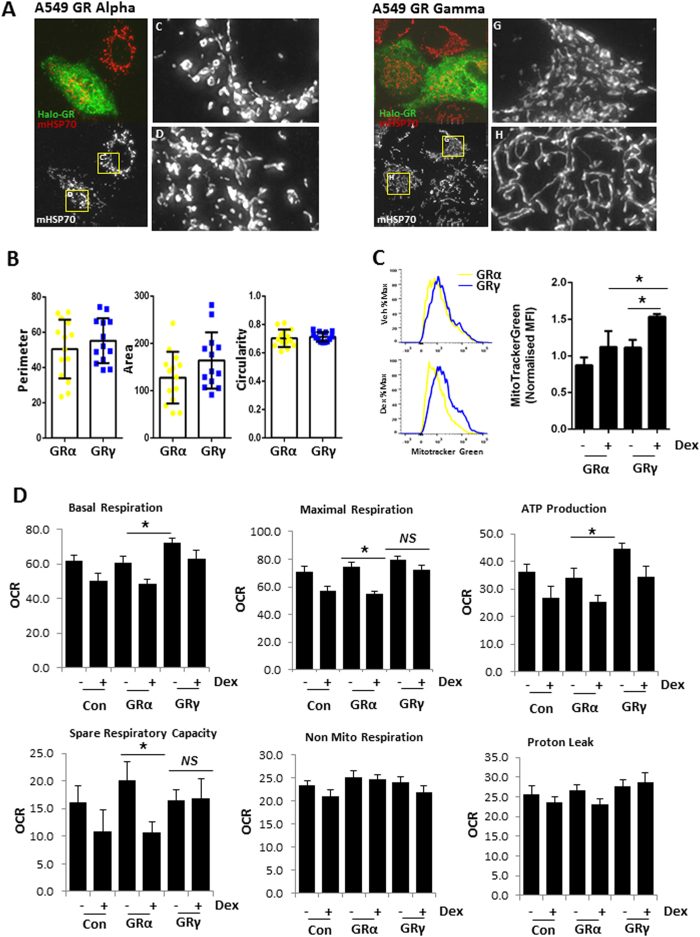
GR regulation of mitochondrial function. (**A**) A549 cells were transfected with 1 μg of either HaloTag GRα or GRγ, then fixed and immunolabelled with antibodies for HaloTag (green) or mitochondrial HSP70 (red). Higher magnification images of boxed regions are shown inset. Representative images are shown. (**B**) Mitochondrial morphology (perimeter, area, circularity) was quantified where each individual data point represents an average across a single cell. Data are presented as the mean ± SD. (**C**) HEK-FlpGRα and HEK-FlpGRγ cells were treated with 100 nM dexamethasone overnight, then incubated with Mito Tracker Green for 1 hr, and analysed by FACS to measure mitochondrial mass. Shown are representative histograms and quantification of mean fluorescence intensity (MFI) for Mitotracker Green staining. Samples compared with one-way ANOVA, and data presented as the mean ± SD of three independent experiments (*p < 0.05). (**D**) HEK-FlpGRα and HEK-FlpGRγ cells were treated with 100 nM dexamethasone overnight, then mitochondrial function measured on a Seahorse XFe96 analyser. Samples compared with one-way ANOVA, and data presented as the mean ± SD of three independent experiments (*p < 0.05). OCR oxygen consumption rate.

As a mitochondrial signal emerged from both transcriptome and protein interactome datasets, we next analysed mitochondrial morphology, mass, and membrane potential. We discovered significant differences in mitochondrial mass but not membrane potential (although there was a trend), or morphology between GRα and GRγ expressing cells (Figs 5A–C, S9 and S10A). Our analysis implied differences in mitochondrial respiration, and so we next investigated this possibility using the Seahorse Xfe analyser (Fig. S10B). In the absence of added ligand, GRγ expressing cells have increased basal respiration and ATP production, compared with GRα expressing cells (Fig. 5D), suggesting a specialised role for GRγ in regulating mitochondrial function.

## Discussion

The GRγ isoform, the result of a constitutive splicing event, is tightly conserved through vertebrate evolution^1^,^2^. However, with the exception of some reports of altered expression in states of Gc resistance^6^,^7^, the GRγ splice variant currently lacks the clear, and unique, biological role required to explain its evolutionary preservation. Here we comprehensively define surprising divergence in function conferred by the single additional arginine, and discover a unique biological role for GRγ.

GRγ takes up a more discretely cytoplasmic localisation than GRα, with striking membrane association, suggesting that GRγ may in fact be the elusive membrane GR which has evaded molecular cloning over the past thirty years^22^,^23^,^24^,^25^,^26^. The membrane association is best appreciated in live cell imaging (movie S2), with clear non-homogeneous, and rapid partition of GRγ to dynamically changing plasma membrane locations. The greater cytoplasmic location is accompanied by slower kinetics of nuclear import, suggesting either increased cytoplasmic tethering, or impaired engagement with the nuclear import machinery. Indeed the inserted arginine is close to one of two nuclear localisation sequences (NLS1). The altered compartmentalisation of GRγ may contribute to altered kinetics of function. Indeed, the delayed onset of GRγ nuclear import was accompanied by a significant delay in transactivation.

Consistent with previous studies, a clear distinction in the pattern of target gene regulation was also seen. The interaction between GRγ and DNA recognition sequences has already shown a distinct difference in sequence specificity rather than affinity^8^, and the overlap in target gene expression adds further to this conclusion; suggesting that binding sites which do not discriminate between GRα, and GRγ are similarly regulated by both^9^. Enrichment analysis revealed an intriguing signal of mitochondrial function as distinguishing between the two GR isoforms.

Our discovery of distinct intracellular locations and trafficking kinetics supports GR isoform specific interaction with distinct protein partners, and so we undertook proteomic analysis of GRα, and GRγ interactomes under basal, and ligand activated conditions. Significant differences were seen under ligand free conditions, providing a clear correlate to the predominantly cytoplasmic location of the GRγ isoform compared to GRα. The cytoplasmic anchoring of GRγ may permit or enhance some of these interactions, and other such interactions may contribute to the anchoring, and delayed translocation kinetics. An important distinction was the mitochondrial interactome engaged by GRγ in the unliganded state. Localisation of the GR to mitochondria has been reported before^27^,^28^,^29^,^30^,^31^, but the earlier analyses did not consider GR isoforms. Our new data suggests that these previous observations result in part from the presence of GRγ.

Identification of GRγ as regulating nuclear genes encoding mitochondrial proteins, and interacting with mitochondrial proteins suggested a coherent biological programme for GRγ in regulating cellular energy metabolism. We analysed mitochondrial morphology, and discovered that both GR isoforms reduced mitochondrial circularity, a measure of mitochondrial fusion^32^.

GRγ expression resulted in a profound increase in mitochondrial mass, when compared to the GRα. Dynamic energy metabolism was investigated further, using Seahorse technology, which revealed GRγ dependent increase in ATP generation, and oxygen consumption. Interestingly, we saw these GRγ specific changes in the absence of added ligand. Therefore, our data supports the identity of complementary, but unique, GR isoform actions on mitochondrial energy expenditure. This also provides a mechanism for the transfer of time of day information to the mitochondria through the HPA axis^33^,^34^. In humans, serum cortisol concentrations are strongly circadian, peaking early morning, falling throughout the day to reach undetectable levels at night. Therefore, at night the ligand independent actions of GRγ on mitochondria would be dominant.

We propose that these differential regulatory properties have permitted the evolutionary conservation of GRγ. Full characterisation of the physiological role of GRγ will require splice site targeting *in-vivo*, but our studies strongly support a unique, and non-redundant role of this isoform in Gc action.

## Materials and Methods

Details of antibodies, plasmids, primers and cells are provided in Supplementary Information.

*Immunoblot analysis*, transfection and reporter gene assays, *quantitative RT-PCR, immunofluorescent microscopy, live cell microscopy and bioluminescent real-time recording* have all been described previously^35^,^36^.

### Transcriptomics

HEK-Flp cells were treated with vehicle or dexamethasone for 4 hrs and RNA extracted and processed using an RNeasy kit (Qiagen). RNA quality was established using an Agilent bioanalyser and duplicate samples analysed by microarray using Affymatrix gene array chips.

A list of Gc regulated genes was generated by stratifying duplicate probe sets for the HEK-Flp cells expressing either GRα or GRγ compared to the HEK-Flp control cells using the Characteristic Direction method^11^. Functional annotation was performed using Enrichr software^12^. Additional information is provided in Supplementary Information.

### Proteomics

A549 cells were transfected with GRα or GRγ (N-terminal Halo-tag) using polyethyleneimine and treated with vehicle or 100 nM dexamethasone for 1 hour. Cells were lysed, DNase treated and incubated with Halo-link resin overnight (4 °C). The resin was washed 6 times with TBS CA-630, and incubated with 30 units of Tobacco Etch Virus (TEV) protease for 2 hours on ice. Samples were electrophoresed, gels stained with Simply Blue Coomasie safe stain and protein bands were excised, destained and dried overnight at 37 °C. Peptides were extracted and loaded onto an Acclaim Pepmap C18 Trap. Analytical separation of the peptides was performed using Acclaim PepMap100C18 Column on a U3000 RSLC (Thermo). Peptides were separated over a 91 minutes solvent gradient on-line to a LTQ Orbitrap Velos (Thermo). Data was acquired using an information dependant acquisiton (IDA) method^37^. Functional annotation was performed using the Enrichr software^12^. Additional information is provided in Supplementary Information.

### Mitochondrial morphology

A549 cells transfected with GRα or GRγ (N-terminal Halo-tag) were stained for Halo-tag and mitochondrial HSP70 as described previously^36^. Mitochondrial morphology was quantified using ImageJ^38^. Deconvolved images were converted into binary images using the default automatic threshold function in ImageJ. Mitochondrial morphology was then quantified, using the built-in ImageJ function Analyze particles to measure the following properties: area, circularity and perimeter.

### Mitochondrial number and membrane potential

HEK- Flp cells were treated with vehicle or dexamethasone 100 nM overnight, then incubated in 50 ng/ml Mitotracker Green (Life Technologies) for 1 hr at 37 °C to measure mitochondrial mass, or 50 ng/ml TMRM (Life Technologies) for 1 hr at 37 °C to measure mitochondrial membrane potential. Cells were trypsinised and staining assessed by flow cytometry, performed on the BD LSR Fortessa cytometer (BD Biosciences). Data was analysed with FlowJo_V10 software (Tree Star).

### Mitochondrial stress assays

HEK-Flp cells were seeded into poly-lysine coated Seahorse culture plates (20 k cells/well) and left to adhere overnight. Cells were treated with vehicle or dexamethasone overnight, then transferred to base media (supplemented with 10 mM Glucose, 1 mM sodium pyruvate and 2 mM glutamine, pH7.4). Mitochondrial stress assays (2 μM Oligomycin, 0.5 μM FCCP) were performed on a Seahorse XFe96 analyser as per manufacturers instructions. Data was analysed using WAVE.

## Additional Information

**How to cite this article**: Morgan, D. J. *et al.* Glucocorticoid receptor isoforms direct distinct mitochondrial programs to regulate ATP production. *Sci. Rep.* **6**, 26419; doi: 10.1038/srep26419 (2016).

## Supplementary Material

Supplementary Information

Supplementary Dataset 1

Supplementary Dataset 2

Supplementary Dataset 3

Supplementary Movie S1

Supplementary Movie S2

Supplementary Movie S3
