# Supplementary material for: Glucocorticoid receptor isoforms direct distinct mitochondrial programs to regulate ATP production
Source: Sci Rep. 2016 May 26;6:26419. doi: 10.1038/srep26419 (PMC4881047; doi:10.1038/srep26419)
Supplement: Supplementary Information [file srep26419-s1.docx]

**Glucocorticoid receptor isoforms direct distinct mitochondrial programs to regulate ATP production.**

David J Morgan ^1,2^, Toryn M Poolman^2,4,5^, Andrew J.K. Williamson^2,5,6^, Zichen Wang^7^, Neil R. Clark^7^, Avi Ma’ayan^7^, Anthony D Whetton^2,5,6^, Andy Brass ^1,3^, Laura C Matthews^2,4,5^*, David W Ray^2,4,5^*

**Supplementary materials and methods (1 file)**

**Supplementary Figures (10 files)**

**Supplementary Movies (3 files)**

**Supplementary Table (1 file)**

**Supplementary Data Files (3 files)**

**Materials:** Anti-hGR (clone 41, BD Biosciences, Oxford, UK); Anti-phospho-(Ser211)-GR, anti αTubulin (Cell Signalling Technology, MA, USA); Anti mHSP70 (Pierce); Phalloidin-TRITC, Mitotracker green, TMRM (Molecular probes); Horseradish peroxidase conjugated anti-mouse and anti-rabbit (GE Healthcare, Buckinghamshire, UK); Dexamethasone, Oligomycin, FCCP, Antimycin A and Rotenone (Sigma, Dorset, UK). AH3-Luciferase. pcDNA5/FRT and pcDNA5/FRT/TO were from Invitrogen. pcDNA5/FRT/GRα, pcDNA5/FRT/GRγ, pcDNA5/FRT/TO/GRα and pcDNA5/FRT/TO/GRγ were generated by subcloning GRα and GRγ from pcDNA3 using BamH1 and Xho1 restriction enzymes. Site directed mutagenesis (QuickChange Lightning Site Directed Mutagenesis Kit, Aglient technologies) was performed on HaloTag-GRα (FHC10483, Promega) to generate HaloTag-GRγ. GRα and GRγ were subcloned into pENT from pcDNA3 using BamH1 and Xho1 restriction enzymes, generating pENT-GRα and pENT-GRγ. pG-EGFP GRα and pG-dsRedXP GRγ were generated using  LR Clonase (Invitrogen), as instructed by the manufacturers, to catalyse a recombination reaction between the appropriate entry and destination vectors. CGT-luc, and KLK3-luc (9) were kind gifts from S. Meijsing (Max Planck Institute for Molecular Genetics, Berlin). Doxycycline (dox; Invitrogen). A list of primers are provided in **Supp. Data File 3**.

**Methods: *Generation of stable cell lines:*** Stable HEK-Flp control, GRα, GRγ and HEK-Flp TRex control, GRα and GRγ cells were generated following cotransfection of either the host HEK-Flp or HEK-Flp TRex cell line with 900ng of a Flp recombinase (pOG44, Invitrogen) and 100ng of one of either pcDNA5/FRT, pcDNA5/FRT/GRα, pcDNA5/FRT/GRγ, pcDNA5/FRT/TO, pcDNA5/FRT/TO /GRα or pcDNA5/FRT/TO/GRγ, respectively. Expression of the Flp recombinase transferred the pcDNA5 cassette and incorporated it into the host cell genome. Positive transfectants were selected through culture with DMEM containing 10% FBS and substituting 0.1% of the mammalian antibiotic Hygromycin (Invitrogen), the media for the HEK-Flp TRex cells line additionally contained blasticidin in a humidified atmosphere of 5% CO_2_ at 37^o^C. HEK-Flp TRex cells were treated with dox (1µg/ml) to switch on the expression of the incorporated pcDNA5 cassette. Using commercially available host cell lines permits homogeneous integration of expression cassettes across the cell population. Pooled transfectants were used for experiments to eliminate effects of clonal selection.

## **Cell culture and maintenance:** Human lung adenocarcinoma epithelial cells (A549; ATCC, Teddington, UK) and human embryonic kidney 293 cell lines that express either a single Flp recombinase recognition site (HEK-Flp, Invitrogen, Paisley, UK) or a single Flp recombinase recognition site and a Tet repressor (HEK-Flp TRex, Invitrogen, Paisley, UK) were cultured in Dulbecco’s modified Eagle’s medium (4500mg glucose/l) (DMEM; Sigma UK) supplemented with 10 % heat inactivated fetal bovine serum (FBS; Invitrogen, Paisley, UK) or 10 % charcoal dextran stripped fetal calf serum (sFCS; Invitrogen). HEK-Flp and HEK-Flp TRex media were both supplemented with 0.1% of the selective antibiotic Zeocin (Invitrogen), while HEK-Flp TRex media additionally contained blasticidin (Invitrogen). All cells were grown in a humidified atmosphere of 5 % carbon dioxide at 37 ^o^C.

***Bioinformatics analysis of the GR transcriptome:*** A list of Gc regulated genes was generated by stratifying duplicate probe sets for the HEK-Flp cells expressing either GRα or GRγ compared to the HEK-Flp control cells using the Characteristic Direction method (11). Functional annotation was performed using the Enrichr software (12).

Briefly, the Characteristic Direction is a geometric approach to identify genes that are differentially expressed between two biological conditions, for example, GRα dexamethasone vs GRγ dexamethasone. Differences between the two conditions can be characterized by a direction in the expression space, which can be represented by a unit vector. The relative magnitudes of the components of this vector correspond to the relative significance of the genes in distinguishing between the two biological conditions. Using this approach we extracted genes that showed a large difference between cell types and grouped them into positive and negative changes and then performed enrichment analysis on these genes using the Enrichr software tool (12).

***Proteomics*:** 1x10^8^ cells were transfected with 30 μg of GRα or GRγ (N-terminal Halo-tag) using polyethyleneimine (90µg) and left for 24 hours. Cells were washed in PBS, the media replaced with serum free DMEM and incubated for 5 hours to remove residual Gc. Following treatment with vehicle or 100nM dexamethasone, cells were washed (PBS), lysed (150 mM NaCl, 0.5% Triton X100, 3 mM MgCl_2_, 20 mM Bicine, 1 µM CaCl_2_ and 1 µM ZnCl_2_ [pH7.4], containing protease and phosphatase inhibitors), passed through a 23-gauge needle and treated with 100 units of DNase (20 minutes, Promega). Cell lysates were cleared and incubated with Halo-link resin (75 µL/mL, prewashed with (TBS) and 0.05% CA-630 (TBS CA-630) overnight (4°C). The resin was washed 6 times with TBS CA-630, re-suspended in 30 µL of TBS CA-630 with 30 units of Tobacco Etch Virus (TEV) protease and incubated for 2 hours on ice. 10 µL of x4 SDS (1% SDS and 50 mM Tris-HCL) elution buffer, 10 µL of Nupage LDS loading buffer and 6 µL of 1M dithiothreitol were added then samples electrophoresed, and gels stained with Simply Blue Coomasie safe stain. Protein bands were excised, destained with repeated incubation in 200 mM ammonium bicarbonate, 40% [v/v] acetonitrile. Gel pieces were dried with three washes in 100% acetonitrile and then trypsinised (Trypsin resuspended in 100 mM ammonium bicarbonate, 5% [v/v] acetonitrile) overnight at 37°C. Peptides were extracted from the gel pieces by incubation in 50% [v/v] acetonitrile, 0.1% [v/v] formic acid, peptides were desiccated and resuspended in 2% [v/v] acetonitrile, 0.05% [v/v] trifluoroacetic acid; pH 2.7. For each analysis, 10% of the peptide sample was loaded onto an Acclaim Pepmap C18 Trap (500 µm x 5 mm) and flow was set to 30 µl/min of 2% [v/v] acetonitrile, 0.05% [v/v] trifluoroacetic acid for 5 min. Analytical separation of the peptides was performed using Acclaim PepMap100C18 Column (3 µm, 75 µm x 500 mm) on a U3000 RSLC (Thermo). Briefly, peptides were separated over a 91 minutes solvent gradient from 2% [v/v] acetonitrile, 0.1% [v/v] formic acid to 40% [v/v] acetonitrile, 0.1% [v/v] formic acid on-line to a LTQ Orbitrap Velos (Thermo). Data was acquired using an information dependant acquisiton (IDA) method (37) where, for each cycle one full MS scan of m/z 300 - 1700 was acquired in the Orbitrap at a resolution of 60,000 at m/z 400 with an AGC target of 106. Each full scan was followed by the selection of the 20 most intense ions, CID and MS/MS analysis was performed in the LTQ. Selected ions were excluded from further analysis for 60 seconds. Ions with an unassigned charge or a charge of +1 were rejected.

Data was analysed using Mascot (Matrix Sciences) the parameters were; Uniprot database, taxonomy *Homo Sapiens*, trypsin with up to 1 missed cleavage allowed, variable modification were oxidised methionine, phosphorylated serine, threonine and tyrosine and the peptide tolerance of 0.025 Da and 0.03 Da for MS/MS tolerance. We performed enrichment analysis on these genes using the Enrichr software tool (12).

**
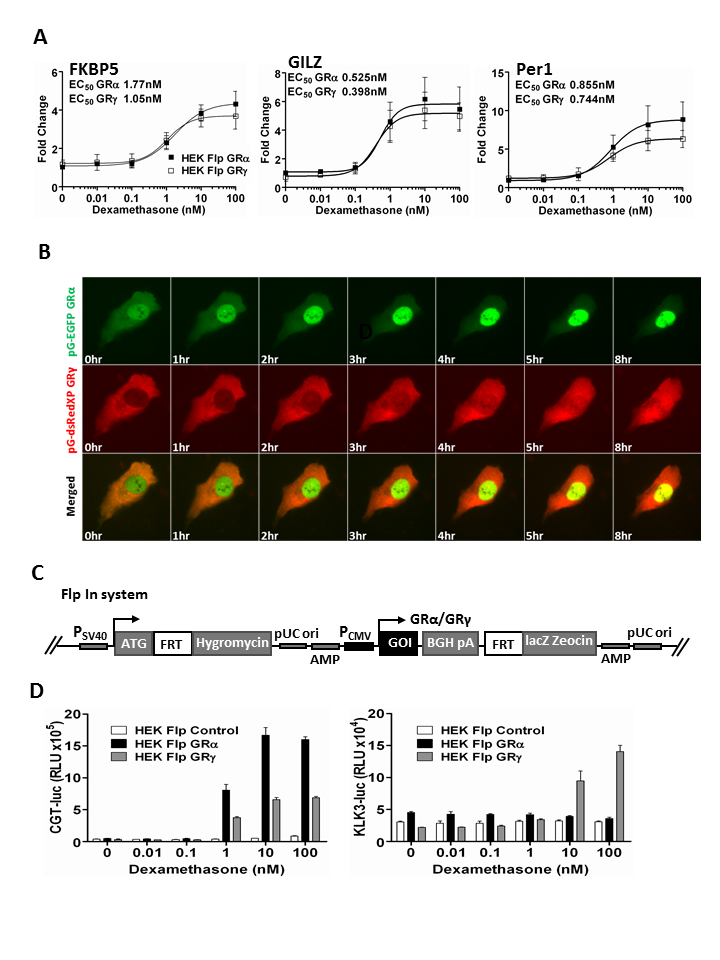
**

**Supplementary Figure 1: Quantification of the translocation kinetics of GRα and GRγ.** (A) HEK-FlpGRα and HEK-FlpGRγ cells were treated with a range of concentrations of dexamethasone (0-100nM), for 4hrs. Cells were lysed, RNA was extracted and analysed by qPCR for the Gc-regulated genes FKBP5, GILZ and PER1. EC50 are shown. (B) Cells were co-transfected with pG-EGFP GRα and pG-dsRedXP GRγ and then treated with 1nM dexamethasone. Nuclear translocation was imaged by time-lapse microscopy. (C) Flp recombinase system enables matched expression and integration site for GR. (D) HEK Flp-In Control, GRα and GRγ cells were transfected with 2μg of either CGT-luc or KLK3-luc. Cells were treated with dexamethasone, then lysed and analysed by luciferase assay.

**
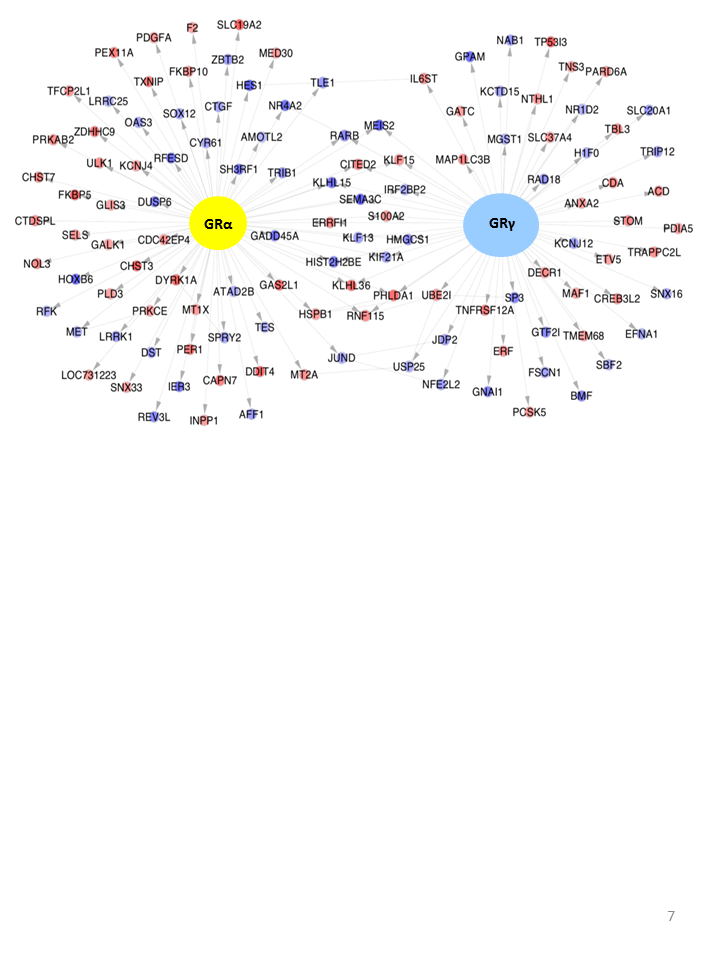
**

**Supplementary Figure 2: Transcription regulatory network by combining the differentially expressed genes identified in this study with a published ChIP-seq dataset for GR.** The differentially expressed genes (DEGs) from comparing GRα or GRγ cells after activation with Dex. GR targets that are significantly up- or down- regulated in response to GR activation are colored in red and blue, respectively. The GR - target relationship were retrieved from ChIP-seq data of NR3C1 in A549 cell line performed in the ENCODE project.

**
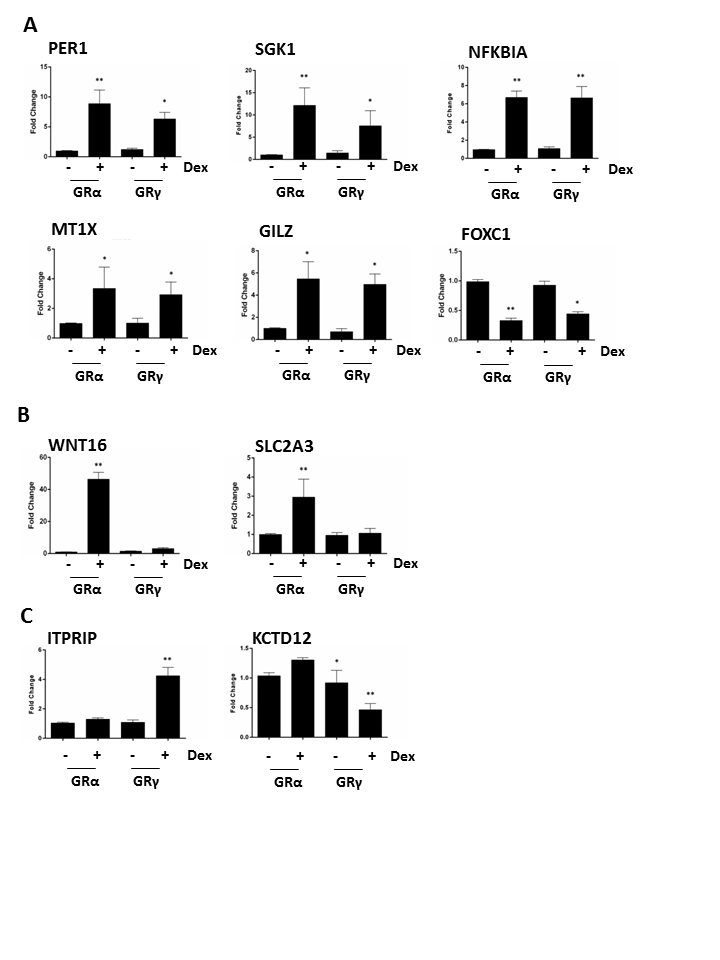
**

**Supplementary Figure 3: HEK-FLP cells Validation**. (A-C) HEK-FlpGRα and HEK-FlpGRγ cells were treated with 100nM dexamethasone for 4 hours, RNA was extracted and cDNA was generated. qPCR was performed to evaluate transcript levels of the panel of gene targets and data analysed by δδ CT method, normalised to a GAPDH control. Graphs (mean ± SD) combine data from three separate experiments and display fold change over vehicle treated control. Stats were performed by one-way ANOVA. Values considered significant when p-value is <0.05. ***** significant compared to vehicle treated controls, ****** significant when compared to all samples. (A) Examples of common GR targets, (B) GRα specific targets and (C) GRγ specific targets are shown.

**
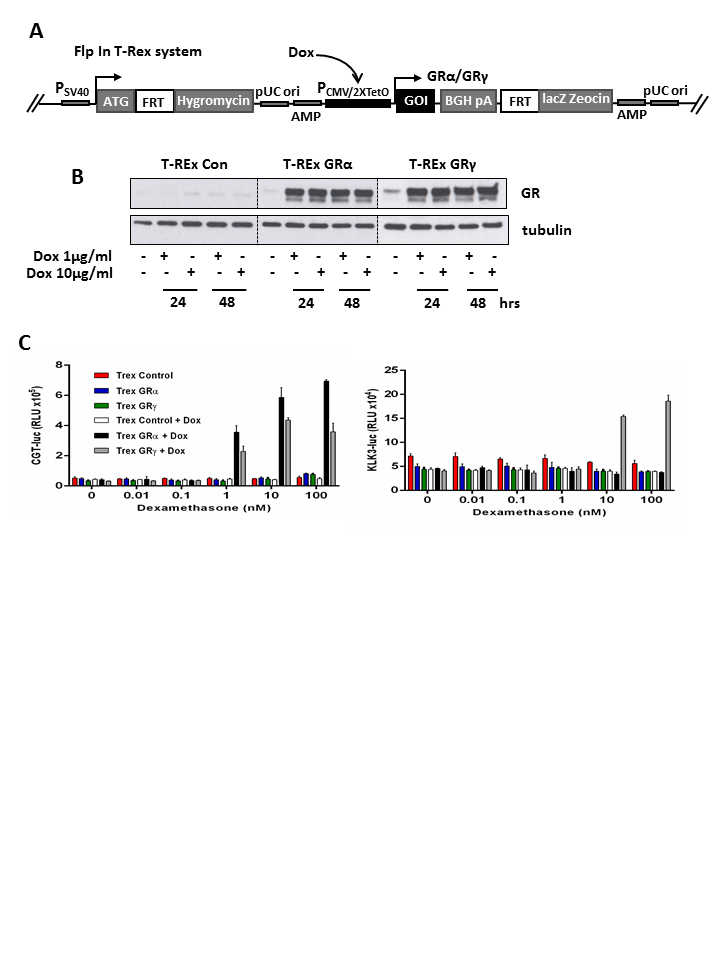
**

**Supplementary Figure 4: T-REx System**. (A) HEK Trex Flp-In system enables matched expression and integration site for GR under the control of tetracycline. (B) HEK Trex Flp-In Control, GRα and GRγ cells were treated with doxycycline (dox) at either 1µg/ml or 10µg/ml for 24 or 48 hours then lysed and analysed by immunoblotting for total-GR and total-tubulin. (C) HEK Trex Flp-In Control, GRα and GRγ cells were co-transfected with 2μg of either CGT-luc or KLK3-luc. Cells were exposed to dox (1µg/ml) 24 hours prior to a 16hr dexamethasone treatment, as indicated, then lysed and analysed by luciferase assay. Graphs depict a single experiment performed in triplicate (mean ± SD).

**
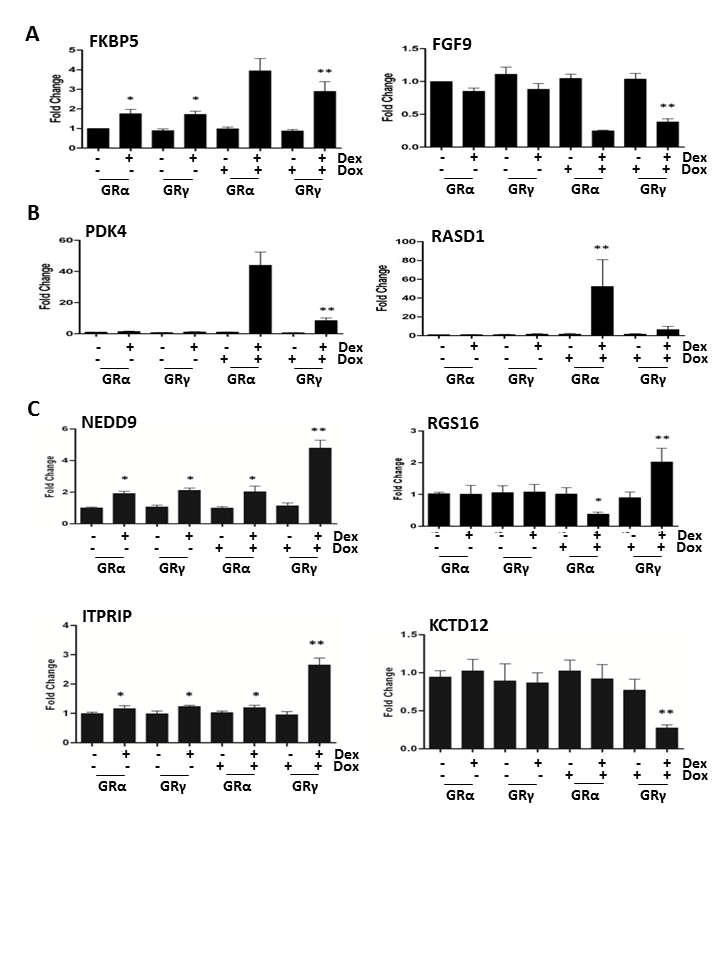
**

**Supplementary Figure 5: Validation of gene array using T-REx System**. HEK Trex Flp-In GRα and GRγ cells were exposed to dox (1µg/ml) 24 hours prior to treatment with 100nM dexamethasone for 4hrs. RT-qPCR was used to assess the expression of shared (A), GRα-(B) and GRγ-(C) specific gene targets. Graphs (mean ± SD) combine data from three separate experiments and display fold change over vehicle treated control. Stats were performed by one-way ANOVA. Values considered significant when p-value is <0.05. ***** significant compared to vehicle treated controls, ****** significant when compared to all samples.

**
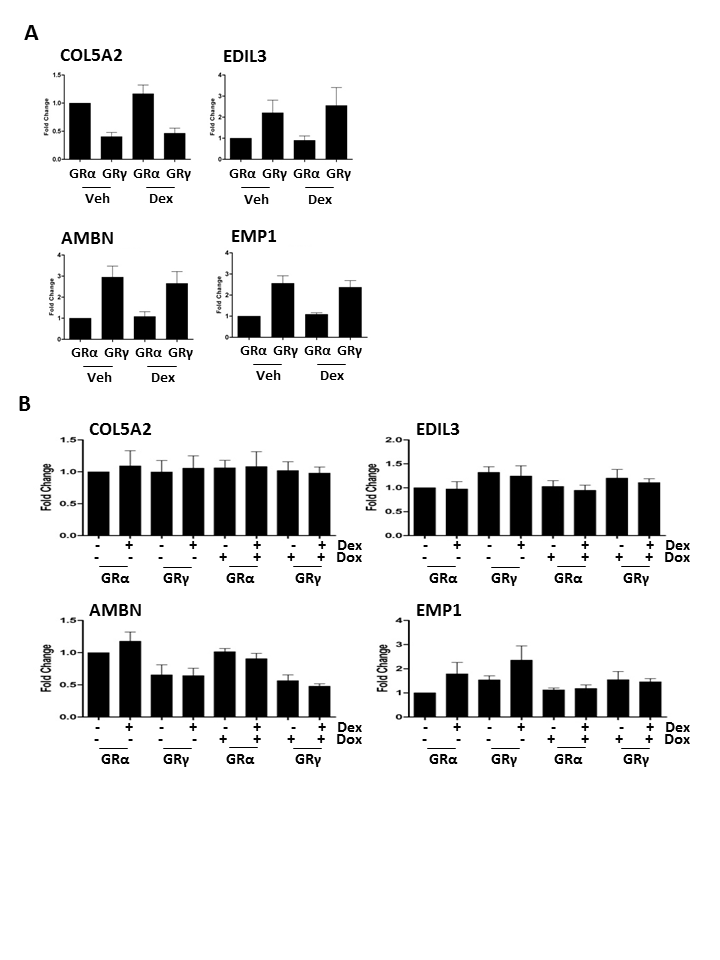
**

**Supplementary Figure 6: Ligand Independent Gene regulation by GRγ.** HEK Flp-In cells (A) and HEK Trex Flp-In cells (B) were treated with 100nM dexamethasone for 4 hours, RNA was extracted and cDNA was generated. qPCR was performed to quantify panel of gene targets and data analysed by δδ CT method, normalised to a GAPDH control. Graphs (mean ± SD) combine data from three separate experiments and display fold change over vehicle treated control.

**
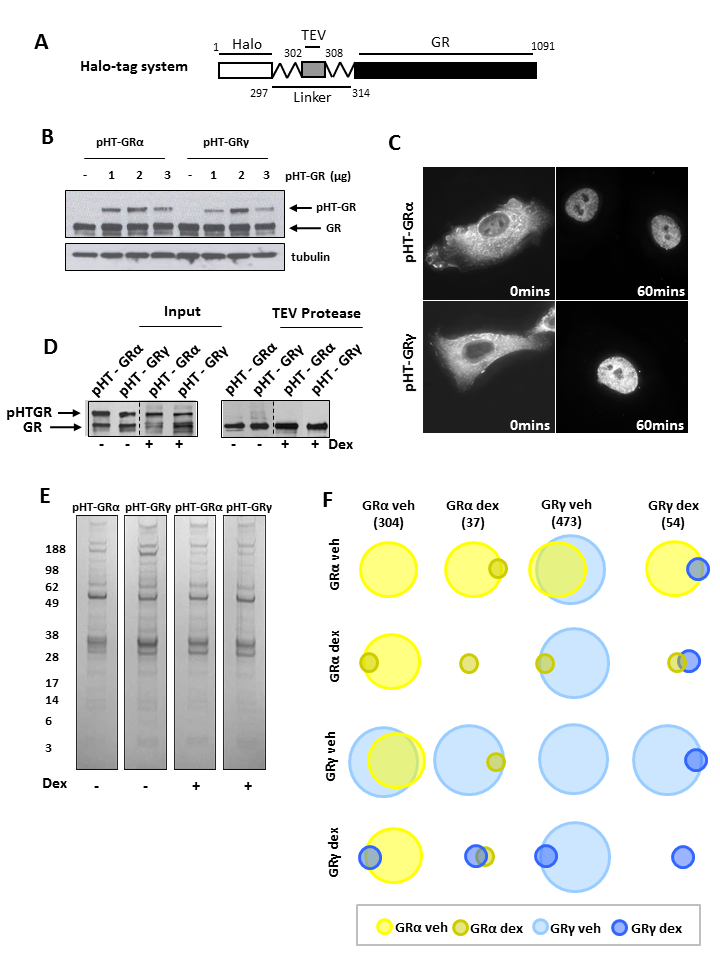
**

**Supplementary Figure 7: Interactome profiling of GRα and GRγ.** (A) Schematic of Halo-tag GR constructs. (B) Transfection of HaloTag GR was optimised using either 0 (-), 1, 2 or 3µg of either construct for 24 hours. Cells were then lysed and analysed by immunoblot for GR and tubulin. (C) To check the subcellular distribution of HaloTag GRα and GRγ, cells were transfected with 1µg of either HaloTag GRα or GRγ and incubated with HaloTag TMRDirect Ligand for 24 hours. Cells were treated with dexamethasone for 1 hour, then fixed and imaged. (D) Input samples and efficient cleaving of Halo-link resin were verified by immunoblot. (E) Purified GR-protein samples were subjected to gel electrophoresis and the gel was stained prior to the excision of the bands and processing of peptides for mass spectrometry. (F) Venn diagram for all GR interacting proteins.

**
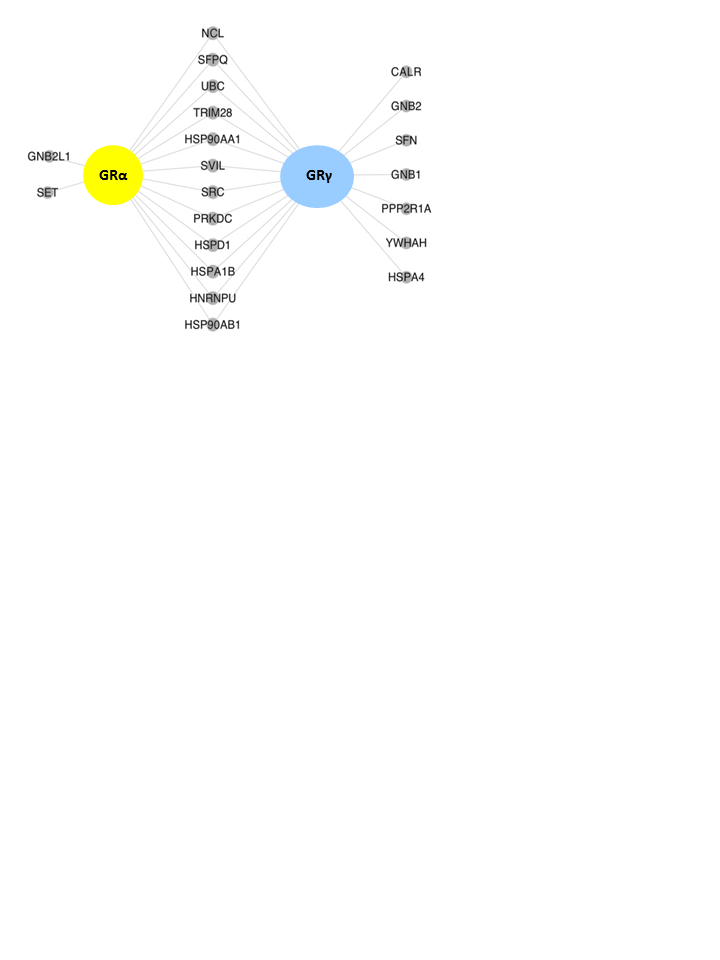
**

**Supplementary Figure 8: Interactome profiling of GRα and GRγ.** Direct protein interactors of GRα and GRγ identified by IP/MS experiment that are confirmed by previously published protein-protein interactions extracted from low-content studies.

**
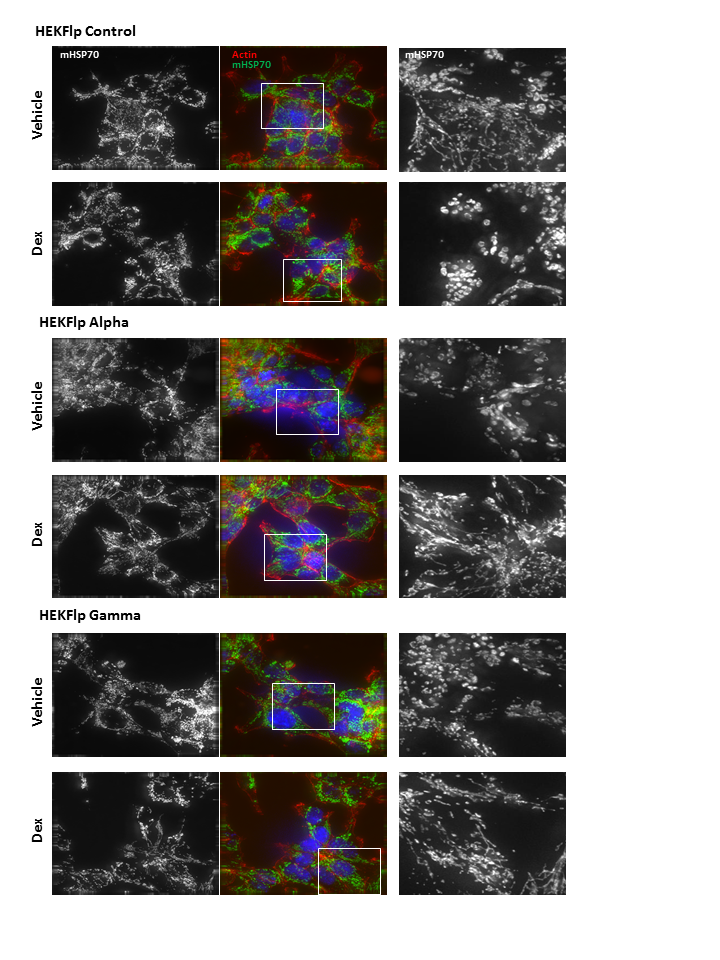
**

**Supplementary Figure 9: GR regulation of mitochondrial morphology**. HEK-FlpCon, HEK-FlpGRα and HEK-FlpGRγ cells were treated with 100nM dexamethasone overnight, then fixed and immunolabelled with an antibody for mitochondrial HSP70 (green), or actin labelled with phalloidin (red). Nuclei were counterstained with Hoechst. Higher magnification images of boxed regions are shown inset.

**
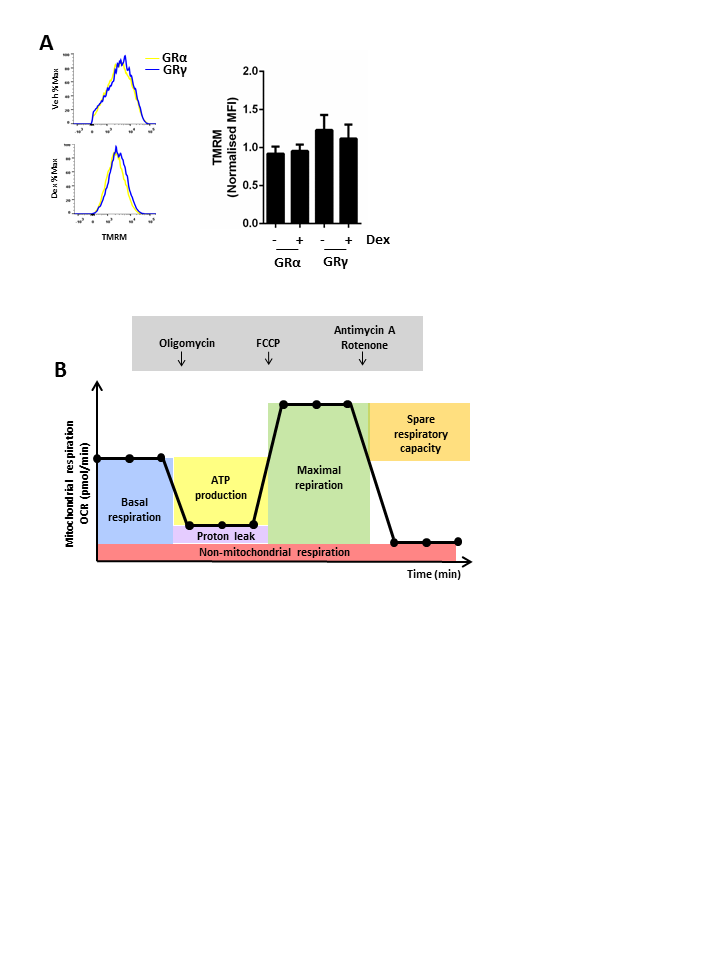
**

**Supplementary Figure 10: Mitochondrial function assay**. (A) HEK-FlpGRα and HEK-FlpGRγ cells were treated with 100nM dexamethasone overnight, then incubated with TMRM for 1hr, and analysed by FACS to measure mitochondrial membrane potential. Shown are representative histograms and quantification of mean fluorescence intensity (MFI) for TMRM staining. Samples compared with one-way ANOVA, and data presented as the mean ±SD of three independent experiments (*p<0.05). (B) Schematic outlining treatment strategy for the mitochondrial stress assay. OCR oxygen consumption rate.

**Supplementary Table 1: Summary of data sets used in this study.** The gene expression gene lists and the proteomics gene lists are from this study. The additional lists are known protein-protein interaction for GR from the literature (LC-PPIs), lists of target genes from ChIP-seq studies, and gene lists associated with mitochondria as listed in the GO cellular component ontology. Each list entry has a URL for Enrichr where the baseline URL is <http://amp.pharm.mssm.edu>/ From these links the enrichment results and the gene sets can be accessed.

| **Gene/protein list** | **Source** | **Size** | **Enrichr Link** |  |
| --- | --- | --- | --- | --- |
| Alpha Dex | Proteomics | 37 | [Enrichr/enrich?dataset=1mb7](http://amp.pharm.mssm.edu/Enrichr/enrich?dataset=1mb7) |  |
| Alpha Vehicle | Proteomics | 304 | [Enrichr/enrich?dataset=1mb8](http://amp.pharm.mssm.edu/Enrichr/enrich?dataset=1mb8) |  |
| GR_alpha_dex-dn | Microarrays | 412 | [Enrichr/enrich?dataset=1mb9](http://amp.pharm.mssm.edu/Enrichr/enrich?dataset=1mb9) |  |
| GR_alpha_dex-up | Microarrays | 185 | [Enrichr/enrich?dataset=1mba](http://amp.pharm.mssm.edu/Enrichr/enrich?dataset=1mba) |  |
| GR_alpha_veh-dn | Microarrays | 301 | [Enrichr/enrich?dataset=1mbb](http://amp.pharm.mssm.edu/Enrichr/enrich?dataset=1mbb) |  |
| GR_alpha_veh-up | Microarrays | 296 | [Enrichr/enrich?dataset=1mbc](http://amp.pharm.mssm.edu/Enrichr/enrich?dataset=1mbc) |  |
| GR_gamma_dex-dn | Microarrays | 316 | [Enrichr/enrich?dataset=1mbd](http://amp.pharm.mssm.edu/Enrichr/enrich?dataset=1mbd) |  |
| GR_gamma_dex-up | Microarrays | 282 | [Enrichr/enrich?dataset=1mbe](http://amp.pharm.mssm.edu/Enrichr/enrich?dataset=1mbe) |  |
| GR_gamma_veh-dn | Microarrays | 292 | [Enrichr/enrich?dataset=1mbf](http://amp.pharm.mssm.edu/Enrichr/enrich?dataset=1mbf) |  |
| GR_gamma_veh-up | Microarrays | 307 | [Enrichr/enrich?dataset=1mbg](http://amp.pharm.mssm.edu/Enrichr/enrich?dataset=1mbg) |  |
| Gamma Dex | Proteomics | 54 | [Enrichr/enrich?dataset=1mbh](http://amp.pharm.mssm.edu/Enrichr/enrich?dataset=1mbh) |  |
| Gamma Vehicle | Proteomics | 473 | [Enrichr/enrich?dataset=1mbi](http://amp.pharm.mssm.edu/Enrichr/enrich?dataset=1mbi) |  |
| LC-PPIs | Literature | 140 | [Enrichr/enrich?dataset=1mbj](http://amp.pharm.mssm.edu/Enrichr/enrich?dataset=1mbj) |  |
| NR3C1-21868756-MCF10A-HUMAN | ChEA | 1132 | [Enrichr/enrich?dataset=1mbk](http://amp.pharm.mssm.edu/Enrichr/enrich?dataset=1mbk) |  |
| NR3C1-23031785-PC12-MOUSE | ChEA | 918 | [Enrichr/enrich?dataset=1mbl](http://amp.pharm.mssm.edu/Enrichr/enrich?dataset=1mbl) |  |
| NR3C1_A549_hg19 | ENCODE | 1855 | [Enrichr/enrich?dataset=1mbm](http://amp.pharm.mssm.edu/Enrichr/enrich?dataset=1mbm) |  |
| NR3C1_ECC-1_hg19 | ENCODE | 237 | [Enrichr/enrich?dataset=1mbn](http://amp.pharm.mssm.edu/Enrichr/enrich?dataset=1mbn) |  |
| NR3C1_HepG2_hg19 | ENCODE | 211 | [Enrichr/enrich?dataset=1mbo](http://amp.pharm.mssm.edu/Enrichr/enrich?dataset=1mbo) |  |
| mitochondrial matrix | GO | 208 | [Enrichr/enrich?dataset=1mbp](http://amp.pharm.mssm.edu/Enrichr/enrich?dataset=1mbp) |  |
| mitochondrial membrane | GO | 457 | [Enrichr/enrich?dataset=1mbq](http://amp.pharm.mssm.edu/Enrichr/enrich?dataset=1mbq) |  |
| mitochondrial respiratory chain | GO | 14 | [Enrichr/enrich?dataset=1mbr](http://amp.pharm.mssm.edu/Enrichr/enrich?dataset=1mbr) |  |
| mitochondrion | GO | 1269 | [Enrichr/enrich?dataset=1mbs](http://amp.pharm.mssm.edu/Enrichr/enrich?dataset=1mbs) |  |

**Supplementary Movie 1: Kinetics of GR translocation**. Cells were co-transfected with pG-EGFP GRα and pG-dsRedXP GRγ, cultured in charcoal stripped serum, treated with 100nM dexamethasone and imaged using time-lapse microscopy.

**Supplementary Movie 2: Localisation of GRγ to membrane ruffles**. Cells were transfected with pG-dsRedXP GRγ, cultured in charcoal stripped serum, and untreated cells imaged using time-lapse microscopy.

**Supplementary Movie 3: Kinetics of GR translocation**. Cells were co-transfected with pG-EGFP GRα and pG-dsRedXP GRγ, cultured in charcoal stripped serum, treated with 1nM dexamethasone and imaged using time-lapse microscopy.

**Supplementary Data File 1: Enrichment P-values microarray.** P-values of the Enrichr analysis performed on the gene expression gene lists from this study.

**Supplementary Data File 2: Enrichment P-values IP/MS.** P-values of the Enrichr analysis performed on the proteomic gene lists from this study.

**Supplementary Data File 3: Primers used for RT-qPCR.** Indicated gene targets were detected using either QuantiTect Primers (Qiagen) or the primer pairs listed.
